# Goat milk with and without increased concentrations of lysozyme improves repair of intestinal cell damage induced by enteroaggregative *Escherichia coli*

**DOI:** 10.1186/1471-230X-12-106

**Published:** 2012-08-11

**Authors:** Eunice B Carvalho, Elizabeth A Maga, Josiane S Quetz, Ila FN Lima, Hemerson YF Magalhães, Felipe AR Rodrigues, Antônio VA Silva, Mara MG Prata, Paloma A Cavalcante, Alexandre Havt, Marcelo Bertolini, Luciana R Bertolini, Aldo AM Lima

**Affiliations:** 1 Department of Physiology and Pharmacology & INCT-Biomedicine, Faculty of Medicine, Federal University of Ceará, Fortaleza, Ceará, Brazil; 2 Department of Animal Science, University of California, Davis, CA, USA; 3 School of Medicine, Health Science Center, University of Fortaleza, Fortaleza, Ceará, Brazil

**Keywords:** Intestinal cells, Cells proliferation, Cells migration, EAEC adherence, Cells viability, Apoptosis, Lysozyme

## Abstract

**Background:**

Enteroaggregative *Escherichia coli* (EAEC) causes diarrhea, malnutrition and poor growth in children. Human breast milk decreases disease-causing bacteria by supplying nutrients and antimicrobial factors such as lysozyme. Goat milk with and without human lysozyme (HLZ) may improve the repair of intestinal barrier function damage induced by EAEC. This work investigates the effect of the milks on intestinal barrier function repair, bacterial adherence in Caco-2 and HEp-2 cells, intestinal cell proliferation, migration, viability and apoptosis in IEC-6 cells in the absence or presence of EAEC.

**Methods:**

Rat intestinal epithelial cells (IEC-6, ATCC, Rockville, MD) were used for proliferation, migration and viability assays and human colon adenocarcinoma (Caco-2, ATCC, Rockville, MD) and human larynx carcinoma (HEp-2, ATCC, Rockville, MD) cells were used for bacterial adhesion assays. Goats expressing HLZ in their milk were generated and express HLZ in milk at concentration of 270 μg/ml . Cells were incubated with pasteurized milk from either transgenic goats expressing HLZ or non-transgenic control goats in the presence and absence of EAEC strain 042 (O44:H18).

**Results:**

Cellular proliferation was significantly greater in the presence of both HLZ transgenic and control goat milk compared to cells with no milk. Cellular migration was significantly decreased in the presence of EAEC alone but was restored in the presence of milk. Milk from HLZ transgenic goats had significantly more migration compared to control milk. Both milks significantly reduced EAEC adhesion to Caco-2 cells and transgenic milk resulted in less colonization than control milk using a HEp-2 assay. Both milks had significantly increased cellular viability as well as less apoptosis in both the absence and presence of EAEC.

**Conclusions:**

These data demonstrated that goat milk is able to repair intestinal barrier function damage induced by EAEC and that goat milk with a higher concentration of lysozyme offers additional protection.

## Background

Diarrheal-causing bacteria can damage intestinal barrier function [1]. Repair of damaged intestinal epithelial cells as a result of bacterial infection requires migration of viable cells adjacent to the site of the damage to cover the area and then cellular proliferation to replace the lost/damaged cells in order to restore normal absorptive and digestive function [1,2]. The process of cell renewal in the intestine therefore involves proliferation, migration, differentiation, apoptosis and necrosis, all of which are related to nutritional status [3,4].

Bacteria can secrete virulence factors and/or adhere to intestinal cells and prevent cellular proliferation and migration, as well as apoptosis and necrosis, four important functions required during the tissue repair process [1]. This is particularly detrimental in young children suffering from diarrhea as diarrheal diseases are a persistent risk factor for malnutrition in many parts of the developing world [5,6]. Damage to the intestinal epithelium leads to a decreased absorptive surface area and less availability of nutrients for growth and development [3,7]. Diarrheal illnesses account for over 1.5 million deaths worldwide each year with bacteria such as enteroaggregative *Escherichia coli* (EAEC) being one of the main causes [8,9]. This type of bacteria can cause growth impairment, malnutrition and cognitive defects even in children not having active diarrhea [7,10]. The pathogenesis underlying the morbidity associated with EAEC is likely due to damage to intestinal barrier function resulting in reduced digestion and absorption of nutrients, altered cell permeability and immune and inflammatory responses [10-12]. However, many questions about the mechanisms remain unanswered.

Oral rehydration solution and breastfeeding are commonly recommended as interventions for diarrheal illnesses [3]. Oral rehydration solution can supply electrolytes to combat the dehydration associated with diarrhea and breast milk can supply key nutrients such as protein and fat as well as several innate antimicrobial proteins such as lysozyme that are thought to help contribute to a healthier gastrointestinal tract [13,14]. One approach to contribute to these current therapies for diarrhea could be the administration of goat milk in general and milk containing enhanced concentrations of lysozyme. Goat is a common source of protein worldwide in the form of both meat and milk. In terms of milk, goat milk is often more tolerable to humans with sensitivities to cows’ milk due to differences in protein and fat composition and structure [15]. For instance, the amino acid composition of goats’ milk is closer to human milk than is cow milk and the fat globules are smaller in goat milk than in cow milk with both factors contributing the greater digestibility of goat milk compared to cow milk.

Concentration of lysozyme in human milk is 1600-3000 times greater than it is in the milk of common dairy animals such as goats and cows [16]. Due to physiological differences, it is not possible to increase the amount of lysozyme in ruminant milk by breeding and selection. We have generated transgenic dairy goats that express human lysozyme (HLZ) in their milk at 68 % the concentrations normally found in human milk [17,18]. The milk displays antimicrobial activity both in vitro and in vivo [19,20] and positively impacts the state of the intestine upon consumption by animal models [21,22]. We hypothesize that the consumption of goat milk and goat milk containing lysozyme could help protect and/or recover damaged intestinal epithelium resulting from bacterial infection and report here the effect of the milks on bacterial adherence and intestinal cell proliferation, migration, viability and apoptosis in the presence of EAEC, a common diarrheal pathogen.

## Methods

### Cell culture

Rat intestinal epithelial cells (IEC-6, ATCC, Rockville, MD) were used for proliferation, migration and viability assays and human colon adenocarcinoma (Caco-2, ATCC, Rockville, MD) and human larynx carcinoma (HEp-2, ATCC, Rockville, MD) cells were used for bacterial adhesion assays. The IEC-6 cells were cultured at 37°C in 5 % CO_2_ in Dulbecco’s modified Eagle medium (DMEM, GIBCO, Grand Island, NY) containing 5 % heat-inactivated fetal bovine serum, 10 mg bovine insulin (SIGMA, St. Louis, MO), 50 U/ml penicillin and 50 μg/ml streptomycin (GIBCO, Grand Island, NY). This cell line comprising intestinal epithelial cells from jejunal crypts of normal rats is commonly used for studies involving intestinal recovery [23]. Caco-2 cells were cultured at 37°C in 5 % CO_2_ in DMEM with 10 % fetal bovine serum, 100 U/ml penicillin and 100 μg/ml streptomycin, non-essential amino acids and sodium pyruvate (GIBCO). Caco-2 cells are frequently used as models of intestinal epithelium as their differentiated monolayers mimic the top of the crypt and base of intestinal villi [24]. The HEp-2 cells were used to standardize the bacterial adhesion assays [25]. Cells were cultured at 37°C in 5 % CO_2_ in minimal essential medium (MEM, GIBCO, Grand Island, NY) containing 10 % fetal bovine serum, 100 U/ml penicillin and 100 μg/ml streptomycin.

### Milk

Goats expressing HLZ in their milk were generated as previously described [17] and express HLZ in milk at concentration of 270 μg/ml [18]. Milk was collected from HLZ transgenic and non-transgenic control goats at the UC Davis Goat Facility of the same breed, parity and stage of lactation. Milk of each type was pooled into respective containers and pasteurized to 74°C. Aliquots were taken and stored at -20°C prior to analysis. Milk was diluted 1:5, 1:20 and 1:40 in DMEM media without glutamine for all assays. All animals were housed and cared for under AAALAC-approved conditions.

### *E. coli* strain

EAEC strain 042 (O44:H18) was kindly provided by Dr. James P. Nataro from the University of Virginia and was used for all assays. This strain was isolated from a child with diarrhea and is considered a standard EAEC strain capable of causing diarrhea in humans [26]. The bacteria were grown on blood agar plates for 18-24 hours then two colonies were diluted in 1 ml DMEM and absorbance at 600 nm recorded. This value became the reference for the standardization of the dilutions required to reach the desired concentration (CFU/ml) of bacteria for use in each assay.

### Cell proliferation assay

Cellular proliferation in the presence of HLZ transgenic and non-transgenic control goat milk was determined using IEC-6 cells (passages 23-27) as previously described [27]. Cells were grown to confluence and then transferred to 96-well plates at a concentration of 4 x 10^4^ cells/ml. Cells were incubated for 48 hours, to approximately 70 % confluence, then the media were removed and replaced with DMEM without glutamine containing 100 μl of HLZ transgenic or control milk at dilutions of 1:5, 1:20 and 1:40. After 24 hours of incubation, 10 μl of the tetrazolium salt WST-1 (4-[3-(4-iodophenyl)-2 H-5tetrazolio]-1-3-benzene disulfonate) was added to each well and incubated for 2-4 hours. WST-1 can be cleaved by mitochondrial dehydrogenase to form formazan, thus the amount of formazan is proportional to the number of metabolically active cells in culture. Formazan concentrations were quantified using an ELISA reader at 450 nm (reference range 420-480 nm).

### Migration assay

The ability of HLZ transgenic and non-transgenic control goat milk to impact cellular migration in the presence and absence of bacteria was determined using IEC-6 cells as previously described [27]. Cells (passages 23-27) were grown to confluence as described above and then transferred to a 12-well plate at a concentration of 2.4 x 10^5^ cells/ml. After 72 hours of growth, cells were incubated with mitomycin C (5 μg/ml) for 15-30 minutes to arrest proliferation. Cells were washed and media replaced with 500 μl DMEM. The monolayer was scratched with a sterile blade to drag the cells to one edge of the well. All wells were then washed twice with DMEM and then incubated in 1 mL of DMEM without glutamine containing HLZ transgenic or control goat milk at dilutions of 1:5, 1:20 and 1:40 in the absence and presence of EAEC-042 at a concentration of 2.5 x 10^5^ CFU/ml. Cells were incubated for 24 hours, washed with HBSS buffer to remove un-adhered bacteria then observed under an inverted microscope at 10X magnification and photographed. The number of cells migrating across the scratch line was counted using Image-Pro Plus (Media Cybernetics, Inc, Bethesda, MD) and calculated based on the surface area of the plate as adapted from McCormack et al. 1992 [23] and Brito et al. 2005 [27].

### Flow cytometry analysis

The effect of HLZ transgenic and non-transgenic control goat milk on the distribution of live, apoptotic and dead cells in the presence of bacteria was determined using IEC-6 cells. Cells (passages 23-27) were grown to confluence as described above then plated in 12-well plates at a concentration of 10^6^ cells/ml [27] and incubated for 24 hours. Media was removed; cells washed and 1 ml of DMEM without glutamine containing HLZ transgenic or control goat milk at 1:5, 1:20 and 1:40 dilutions and 2.5 x 10^5^ CFU/ml EAEC-042 was added. After 24 hours of incubation, media was removed and reserved and cells were trypsinized and added to the reserved media. The cell suspension (135 ml) was incubated with 5 ml of 7-amino-actinomycin (ADD) to stain necrotic cells and 10 ml of Annexin-V-PE to detect apoptotic cells by binding to externalized phosphatidylserine. After 20 minutes of incubation on ice, the samples were analyzed by flow cytometry (EasyCyte, Guava Technologies, USA) with red and yellow filters.

### Bacterial adhesion

The ability of EAEC-042 to adhere to intestinal cells in the presence of HLZ transgenic and control goat milk was determined using Caco-2 cells. Cells (passages 45-49) were seeded into 24-well plates at a concentration of 5 x 10^5^ cells per well and differentiated for a period of 14 days. The media was then replaced with glutamine-free DMEM containing HLZ transgenic and non-transgenic control goat milk at dilutions of 1:5, 1:20 and 1:40 and 2.5 x 10^5^ CFU/ml of EAEC-042. After 1.5 hours of incubation, non-adherent cells were removed by washing with HBSS buffer and then lysed with 1 % Triton-X 100. A total of 100 μl of the lysate was spread on MacConkey agar plates and colonies quantified by counting after 24 hours of incubation at 37°C [28]. As the standard test cell line for EAEC adherence, HEp-2 cells (passage 8) were used to confirm EAEC adherence ability. Cells were placed in 8-well glass slides at a concentration of 5 x 10^4^ cells/ml. After 24 hours of incubation with the monolayer at approximately 50 %, the media was removed and replaced with 400 μl of HLZ transgenic or non-transgenic control goat milk at 1:5, 1:20 and 1:40 dilutions prepared in DMEM without glutamine and 0.2 x 10^2^ CFU/ml of EAEC-O42. After 3 hours of incubation at 37°C, the cells were washed and then stained with May-Grunwald Giemsa and photographed under the microscope.

### Statistical analysis

All assays were performed in triplicate and data reported as the mean ± SEM (standard error of the mean). Data were compared using nonparametric multivariate analysis ANOVA followed by the Bonferroni test using GraphPad Prism software (version 4.01). *P* values less than 0.05 were considered significant.

## Results

### Proliferation

**Figure 1:**
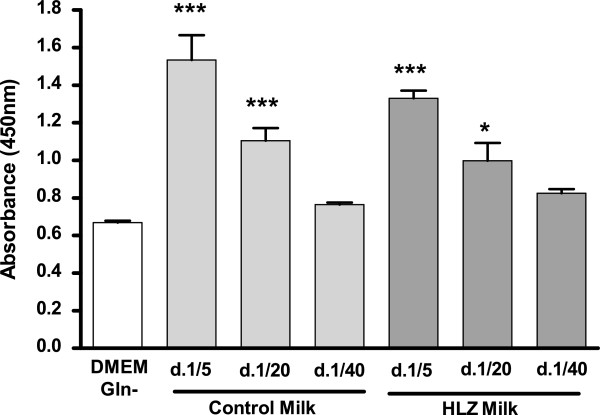
**HLZ and control goat milk enhance IEC-6 proliferation.** Proliferation of IEC-6 cells after 24 hours of incubation with milk from HLZ transgenic or non-transgenic control goats at dilutions of 1:5, 1:20 and 1:40. HLZ and control milk was significantly different from glutamine free DMEM medium (DMEM Gln-), *P < 0.05; *** *P* < 0.001.

Cellular proliferation was significantly greater in the presence of both transgenic and control goat milk at both the 1:5 and 1:20 dilutions compared to cells with no milk (Figure 1). Control milk at a 1:5 dilution had significantly more proliferation than transgenic milk at the 1:20 and 1:40 dilutions (*P* < 0.001). Transgenic milk at a 1:5 dilution had significantly more proliferation than the highest dilution (1:40) of control milk (*P* < 0.001).

### Migration

**Figure 2:**
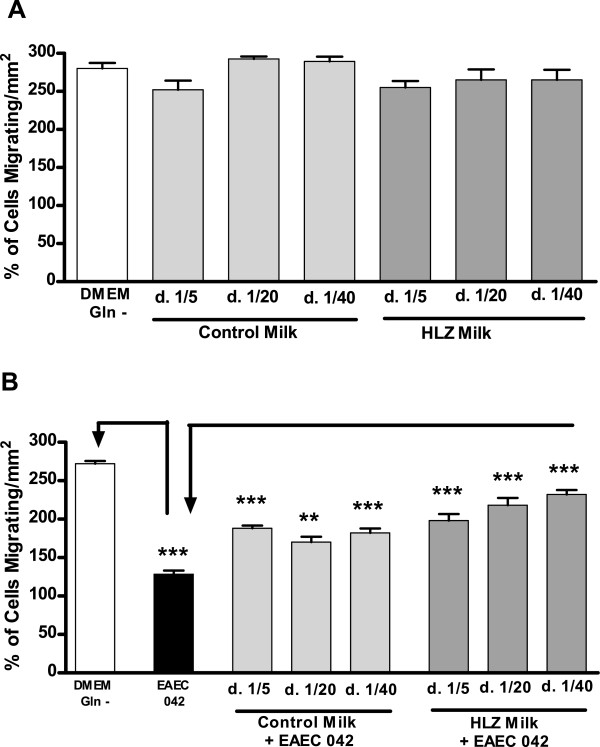
**HLZ and control goat milk improve cellular migration in the presence of EAEC.** Migration of IEC-6 cells after 24 hours of incubation with milk from HLZ transgenic or non-transgenic control goats at dilutions of 1:5, 1:20 and 1:40 in the absence (**A**) and presence (**B**) of EAEC-042 (2.5 x 10^5^ CFU/ml) (**P < 0.01; *** *P* < 0.001).

The migration of IEC-6 cells was not significantly impacted by the presence of transgenic or control goat milk in the absence of bacteria compared to media alone (Figure 2a). Transgenic milk at a 1:40 dilution had significantly more migration than did control goat milk at the lowest dilution of 1:5 (*P* < 0.01). In the presence of bacteria (EAEC-042), migration was significantly lower compared to cells without bacteria when incubated in media alone (*P* < 0.001) but significantly higher when incubated with HLZ transgenic or control milk (Figure 2b). Milk from HLZ transgenic goats at a 1:20 dilution had significantly more migration compared to control milk at both 1:20 (*P* < 0.001) and 1:40 (*P* < 0.05) dilutions and HLZ milk at the highest dilution (1:40) had significantly more migration than any of the control milk dilutions (*P* < 0.001).

### Adhesion

**Figure 3:**
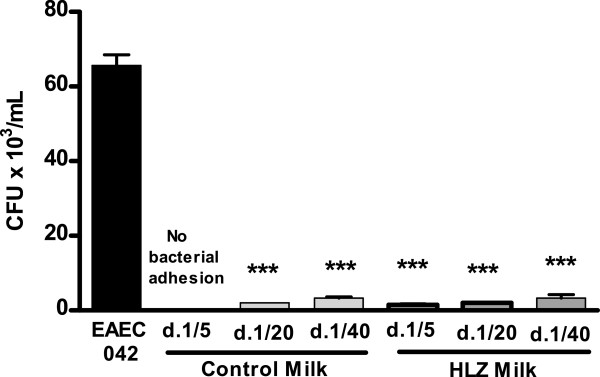
**HLZ and control goat milk decrease bacterial adhesion.** Number of colony forming units of EAEC 042 (2.5 x 10^5^ CFU/ml starting concentration) that adhered to Caco-2 cells after 1.5 hours of incubation with milk from HLZ transgenic and non-transgenic control goats at dilutions of 1:5, 1:20 and 1:40 (*** *P* < 0.001).

**Figure 4:**
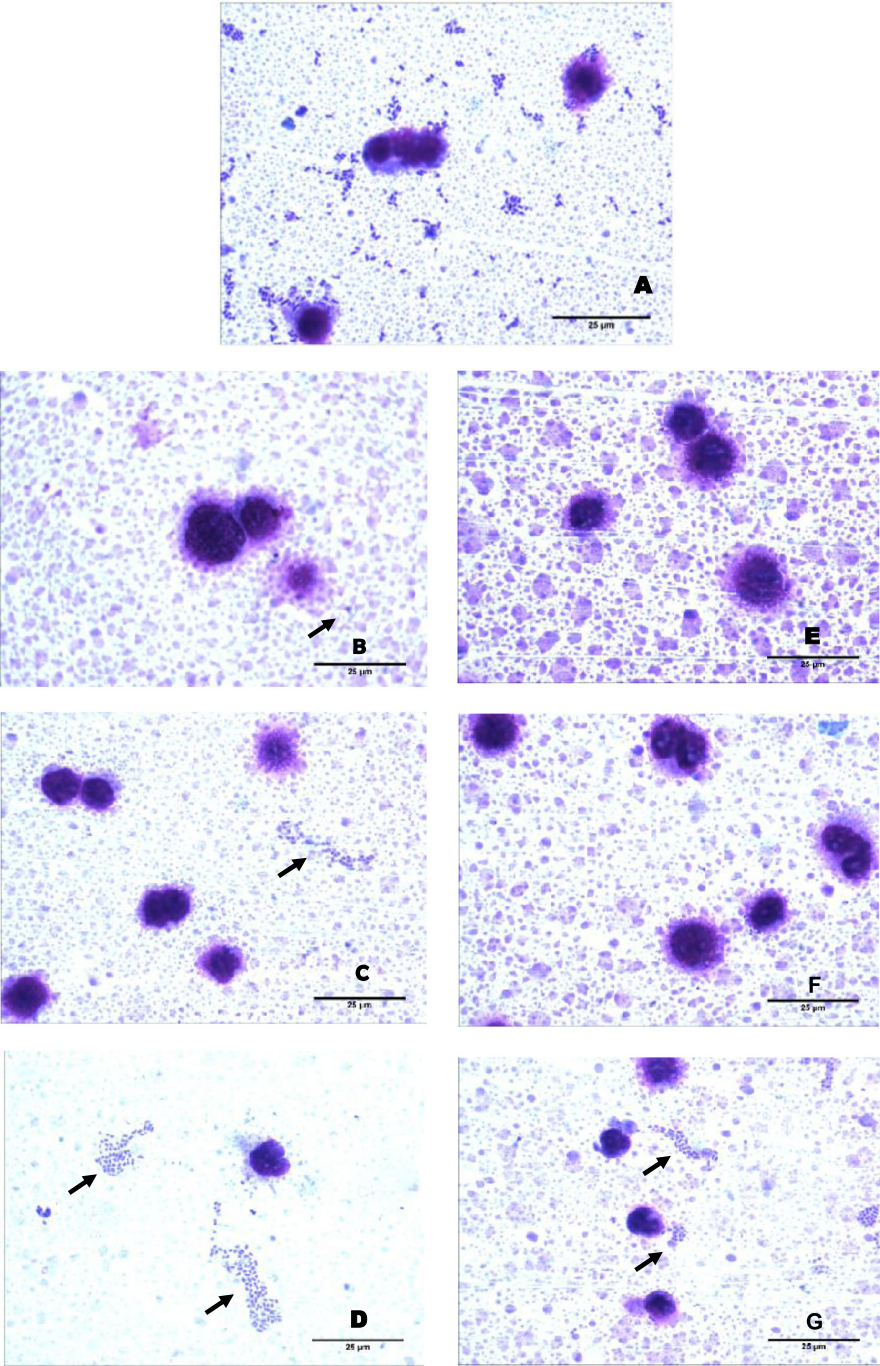
**Bacterial adhesion in HEp-2 cells or in the media their self as indicated by the black arrow.** (**A**) EAEC 042 (0.2 x 10^2^ UFC/mL); EAEC 042 plus milk from non-transgenic control goats at a (**B**) 1:5, (**C**) 1:20 and (**D**) 1:40 dilution; EAEC 042 plus milk from HLZ transgenic goats at a (**E**) 1:5, (**F**) 1:20 and (**G**) 1:40 dilution. 1000X magnification.

There was a significant reduction in the adhesion of EAEC-042 to Caco-2 cells in the presence of both milks (Figure 3). There were no significant differences between control and HLZ milk. In assays using HEp-2 cells, some qualitative differences could be seen between the milk types with transgenic milk having less colonization (Figure 4).

### Cellular viability

**Figure 5:**
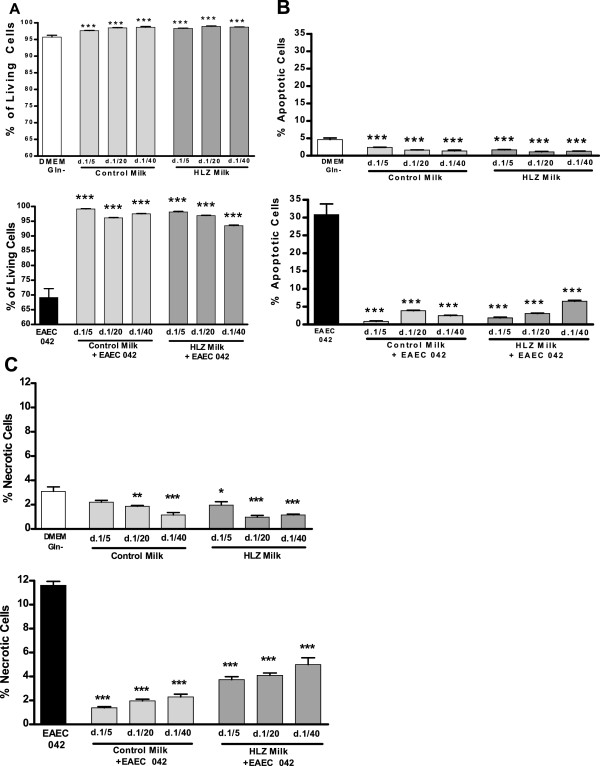
**HLZ and control goat milk improve cellular viability.** Flow cytometry analysis was conducted to determine the percentage effect of HLZ or control milk on (**A**) living IEC-6 cells in the absence and presence of EAEC-042; (**B**) apoptotic IEC-6 cells in the absence and presence of EAEC-042; and (**C**) necrotic IEC-6 cells in the absence and presence of EAEC-042. The IEC-6 cells were incubated for 24 hours in HLZ transgenic or non-transgenic control goat milk at dilutions of 1:5, 1:20 and 1:40 in the absence or presence of EAEC-042 (2.5 x10^5^ CFU/ml) (*** *P* < 0.001).

Both HLZ transgenic and non-transgenic control goat milk significantly increased cellular viability in the absence and presence of EAEC-042 (Figure 5a). There was a correlating effect on viability with transgenic milk at the highest dilution (1:40) having the lowest viability. The percentage of cells in apoptosis and necrosis were also significantly reduced both in the absence and presence of EAEC-042 (Figures 5b and 5c, respectively). As with viability, there was a correlation between dilution and effect with the more diluted milk having more apoptotic and dead cells.

## Discussion

Morbidity and mortality from diarrheal illnesses remains a burden worldwide. While not able to prevent diarrhea, oral rehydration solution can reverse the fluid loss and dehydration responsible for a majority of the deaths due to diarrhea and remains the main treatment for this condition [29]. More recent work has demonstrated that supplementation with micronutrients such as zinc, glutamine and alanyl-glutamine can improve outcomes [30-33]. However, the mechanisms involved in these biologic effects remain poorly defined. As a potential alternative or addition to oral rehydration therapy, we report here the impact of goat milk with and without increased concentrations of the human milk antimicrobial protein lysozyme on intestinal barrier function in vitro.

We conducted several assays which demonstrated the mitigation of the effects of EAEC with both goat milk and goat milk containing increased concentrations of lysozyme. In the absence of injury, both HLZ transgenic and control goat milk stimulated cellular proliferation in IEC-6 cells compared to cells with no milk supplementation. Milk is known to be beneficial to growth and these data demonstrate that goat milk in general can be beneficial to intestinal cell proliferation. Both protein and fat are required nutrients for proper cellular functioning and it is likely that the presence of these ‘extra’ nutrients contributed to the proliferation of the intestinal cells in culture. These data also indicate that the presence of lysozyme in milk does not directly enhance proliferation in its own. It should be noted that these assays conducted with milk without fat resulted in no differences (data not shown) pointing to the presence of the fat as an important component.

When cell injury was induced by the scraping of the cell monolayer, the presence of milk did not significantly improve migration in the absence of bacteria, however, in the presence of EAEC-042 milk significantly improved cell migration with HLZ transgenic milk performing better than control goat milk. Nutrients in the milk, particularly the fat, could be acting to prevent the EAEC from associating with the intestinal cells thus allowing them to maintain migration. Goat's milk has a high content of short chain fatty acids (caprylic, capric and caproic) that are important in reversing poor dietary intake and intestinal disorders [34] and have been found to act as antimicrobial agents [35,36]. The enhanced effect by the lysozyme-containing milk from transgenic animals over non-transgenic control milk may be due to the antimicrobial activity of lysozyme. Previous work has demonstrated that there were no significant differences in the overall amount of fat in HLZ milk compared to control milk [18] and that the protein composition of the two milks differ only in the presence of lysozyme [37]. The HLZ milk has been shown to have antimicrobial activity against several microorganisms including *E. coli* both in vitro [19] and at the level of the intestine [20,21]. However, purified lysozyme alone or added to control milk was not as efficacious an antimicrobial agent as the HLZ milk itself [19], indicating that the in vivo-produced HLZ is more potent. It is therefore likely that lysozyme is exerting antimicrobial activity to reduce the number of EAEC present, although this could not be quantified with this assay. Upon intestinal infection, certain bacteria can adhere to the intestinal wall and cause damage to the intestinal epithelium. Thus, the maintenance of the epithelial barrier is essential for proper functioning and prevention of the entry of pathogenic bacteria leading to an inflammatory response [38]. Both control and transgenic goat milk significantly reduced bacterial adhesion with HLZ milk offering no advantage over control milk. In this assay, cells were incubated with milk for only 1.5 hours, likely too little time for lysozyme to act in a significant antimicrobial fashion. The mechanism of pathogenesis of EAEC infection involves the adherence of EAEC-042 to the mucosal surface of the intestine via aggregative adherence fimbriae (AAF), secretion of toxins and mucosal inflammation by the induction of interleukin 8 (IL-8) release [39]. IL-8 plays an important role in the pathogenesis of EAEC, as it recruits neutrophils to the intestinal epithelial mucosa causing destruction of the epithelium and promoting fluid secretion [40]. This may be related to atrophy of the villi with reduced digestion and absorption of nutrients, one of the mechanisms that could explain weight loss in affected malnourished children. While HLZ milk offered no distinct advantage over control milk in this assay, previous work in vivo has demonstrated increased villi height and expression of the anti-inflammatory cytokine TGF-β upon consumption of HLZ milk indicating that the milk has a protective effect on the intestinal epithelium [21,22] and combined with this data suggests a likely beneficial clinical impact on EAEC infection.

Evidence of altered intestinal barrier function in the presence of EAEC-042 was seen as there was a reduction in IEC-6 viability accompanied by an increase in apoptosis (2 times greater than control) and necrosis (5 times greater than control). When supplemented with milk from HLZ transgenic and non-transgenic control goats, these effects were mitigated. Again, the nutrients in the milk may be protecting the cells from the effects of the bacteria and enhancing their viability. Further studies are required to elucidate the mechanisms involved. Cell viability of IEC-6 cells with induced oxidative damage was increased by the addition of human colostrum, but not cow milk or infant formulas, and the increase was attributed to the polyamine spermine reducing the oxidative stress [41]. Polyamines are small cationic molecules that are found in human milk and are required for intestinal growth and development [42]. Lysozyme also carries a net positive charge and is a relatively small protein (14 kDa) and it is likely that properties of lysozyme other than its antimicrobial function are contributing to the effect of lysozyme-rich milk. Further work is required to dissect out the role of lysozyme on these various aspects of gut barrier function.

## Conclusions

This study addressed the implications of milk consumption at the level of the intestine, in which much is unknown about the role of milk on the intestinal epithelium. Results of these in vitro assays demonstrate that goat milk can counteract the effects of EAEC on cell renewal and repair and intestinal cell barrier function and that goat milk containing higher concentrations of lysozyme offers additional protection. More studies are warranted regarding the mechanism of action of milk and lysozyme on intestinal function with hopes that this could be an additional approach to help fight diarrhea.

## Competing interests

The authors declare that they have no competing interests.

## Authors’ contributions

EC, JQ, IL, HM, FR, AS, MP, and PC: Participated in the in vitro experiments, performed data analysis and drafted the manuscript. EM, MB, and LB: Participated in the in vivo source of goat milk with and without increased concentrations of lysozyme, analysis and helped drafting the manuscript. AH and AL: Participated in the study design, analysis and writing the paper. All authors read and approved the final manuscript.

## Pre-publication history

The pre-publication history for this paper can be accessed here:

http://www.biomedcentral.com/1471-230X/12/106/prepub
